# Fetal Diagnostics using Vision Transformer for Enhanced Health and Severity Prediction in Ultrasound Imaging

**DOI:** 10.2174/0115734056360199250227053012

**Published:** 2025-03-17

**Authors:** Eshika Jain, Pratham Kaushik, Vinay Kukreja, Sakshi, Ayush Dogra, Bhawna Goyal

**Affiliations:** 1 Centre for Research Impact & Outcome, Chitkara University Institute of Engineering and Technology, Chitkara University, Punjab, India; 2 Amity Institute of Information Technology, Amity University, Noida, India; 3 Marwadi University Research Centre Department of Engineering, Marwadi University, Rajkot, Gujarat, India; 4 Faculty of Engineering, Sohar University, Sohar, Oman

**Keywords:** Fetal health classification, Ultrasound images, Transformer, Severity detection, Prenatal diagnosis, Fetal anomaly detection

## Abstract

**Aim:**

This research aims to develop and evaluate a novel health classification and severity detection system based on Vision Transformers (ViTs) for fetal ultrasound imagery. This contributes to improved precision in fetal health status detection and abnormalities with more accurate results than other traditional models.

**Background:**

Amidst the other imperatives of resource-deficient developing nations, mitigating neonatal mortality rates is a challenge that demands precision-based solutions in the era of artificial intelligence. Though the advent of machine learning models has added an optimal dimension to deal with emerging complexity in fetal ultrasound imagery, there is a call to address the huge gap in the demanded precision for prediction than the existing interpretation.

**Objective:**

This research strives to formulate and access a novel health classification and severity detection system based on the implementation of the Vision Transformers frameworks. This pioneering investigation represents an unparalleled exploration into the efficacy of ViTs for discerning intricate patterns within fetal ultrasonographic imagery, facilitating precise categorization of fetal well-being and prognosticating the magnitude of potential anomalies.

**Methodology:**

A private and confidential dataset of 500 fetal ultrasound images has been collected from diverse hospitals. Each image has been annotated by radiologists according to two main labels: the health status of the fetus, which includes healthy, mild, moderate, or severe, and the severity of abnormalities as a continuous measure. At different levels, the dataset underwent pre-processing via distinct techniques. Then, the composite loss function Cross-Entropy has been deployed to train the optimized VIT model using the Adam algorithm.

**Results:**

The classification accuracy of the proposed model is 90% for detecting the severity with an F1-score of 0.87 and MAE of 0.30. The research ascertained that the model ViT evinced a superlative efficacy for the capturing of fine-grained spatial relations in ultrasound images to produce revolutionary predictions.

**Conclusion:**

These results emphasize that ViTs have the potential to revolutionize fetal health monitoring and will contribute significantly to reducing neonatal mortality by supplying clinicians with accurate and reliable predictions for early interventions. This work stands as a yardstick for further diagnostic applications using AI in fetal health care.

## 1 INTRODUCTION

### 1.1 Fetal Assessment: A Cornerstone of Comprehensive Prenatal Care

Fetal assessment forms one of the cornerstones of modern prenatal care. The process is essential not only for monitoring the ongoing health and development of the fetus but also for safeguarding the mother's well-being. The systematic assessment of the fetus, apart from being regular in prenatal visits, is also multifaceted. It enlists the various diagnostic tools and procedures, which were all designed to furnish comprehensive insights into the condition of the fetus throughout the pregnancy. Predominantly, the purpose of fetal assessment is to follow up on the development of a fetus and, hence, delineate any complications at the onset. It gives the healthcare provider an opportunity to take necessary measures for the improvement of outcomes for both the mother and the baby. Fetal assessment can be as simple as routine follow-up or as complex as a special test depending on the risk factors of pregnancy and the development of concerns at any time during the gestation period. Infants are assessed by ultrasound imaging, including Doppler studies, non-invasive prenatal testing for genetic anomalies, and monitoring of the fetal heart rate. These technologies now permit clinicians to see an image of the fetus, monitor the growth patterns of the fetus, review the development of the organs, and track blood flow and heart functioning [1]. Each tool contributes vital information that fills in an accurate picture of the health of the fetus, thereby allowing healthcare professionals to uncover even very serious problems, such as congenital disabilities, growth restrictions, or signs of fetal distress. As prenatal care has continued to evolve, so has the complexity of fetal assessment, hence becoming an intrinsic part of pregnancy management, especially in those that are considered “high-risk.” Examples are if the mother has pre-existing health problems, such as diabetes or hypertension, or if she has had problems in previous pregnancies; in such cases, fetal assessments are even more critical. Assessments offer a chance for early identification and appropriate interventions that may prevent or mitigate the problems. Fetal assessment is not only monitoring the fetus but also gives some insight into clinical decision-making. Information from such assessments aids in making decisions on timing and mode of delivery, further testing if necessary, and interventions [2]. The information provided in high-risk pregnancies may be lifesaving as such information guides clinicians to act in time to prevent such severe outcomes for both mother and baby [3]. Besides its clinical value, fetal assessment gives a sense of assurance to the expecting parents. Being able to see the baby and receive frequent status reports on the baby's condition can help diminish most of the apprehensions of pregnancy. This reassurance is even more valid in pregnancies already complicated by earlier losses or health issues, where ongoing monitoring will be of vital emotional support to the parents. In a nutshell, fetal assessment is dynamic and an integral part of prenatal care. Finally, it provides healthcare providers with the tools and information they need to monitor fetal growth for early detection of problems, which will inform clinical decisions targeted at ensuring the best possible outcomes [4]. The importance of accurate and timely fetal assessment will continue to increase as prenatal care continues to advance, cementing this modality as one of the cornerstones in maternal and fetal health [5].

### 1.2 Fetal Health: The Importance of Monitoring

Continuous fetal health monitoring has been advanced as one of the cornerstones of prenatal care for achieving optimum pregnancy outcomes. Primarily, fetal health monitoring aims at the early detection of a possible complication such as IUGR, congenital anomalies, or any manifestation of fetal distress [6]. Early detection is of prime importance because appropriate interventions could be initiated by healthcare providers well in time, thereby reducing risks of maternal and neonatal morbidity and mortality. The hallmark of contemporary prenatal care is that it is progressively dependent on quality data for clinical decisions [7]. Therefore, the need for accurate and timely assessment of fetal health has been practically paralleling the advancement of medical technology. Clinicians today may employ a set of advanced diagnostic modalities, which include high-resolution ultrasound, Doppler studies, non-invasive prenatal testing, and electronic fetal monitoring. Each of these tools casts light on different aspects of health, and thus, all together, they might give a full picture of the condition of the fetus. High-resolution ultrasound enables the stunning visualization of fetal anatomy in great detail, whereby structural anomalies can be diagnosed well in advance [8]. Another modality, Doppler ultrasound, assesses blood flow through fetal vessels and may be indicative of any possible compromise in fetal oxygenation or circulation. NIPT, on the other hand, offers a non-invasive means to screen for chromosomal abnormalities, offering a way into important genetic information without the risks associated with invasive procedures like amniocentesis [9]. These tools provide current information that is greatly valued in making clinical decisions. For instance, when restrictions to fetal growth are detected, the clinicians have to monitor the situation closely and make appropriate decisions, whether in the adjustment in maternal care or planning of an early delivery. In this regard, timely interventions such as cesarean delivery may be life-saving for the fetus to prevent further complications, which would put both mother and baby in danger. Besides, the continuous development in technologies of fetal health monitoring has made personalization a core method of prenatal care [10]. Clinicians are going to integrate various kinds of diagnostic data to create a personalized plan of care for each pregnancy. In caring for a high-risk pregnancy, there is little room for error and a great need for precise monitoring. As fetal health monitoring technologies continue to evolve, their impact on prenatal care is becoming increasingly important. These are improvements not only in the accuracy of diagnosis but also in the outlook for detection and management capability in pregnancy. The improved overall quality of this technology raises the standard of maternal and neonatal outcomes in various patient populations [11]. In all, continuous monitoring of fetal health is indefatigably important. Overall care for the fetus, early complication detection, and timely and effective intervention are just to name a few reasons why continuous prenatal care is very important. As medical technology continues to improve, the ability to monitor fetal health with increasing precision will further improve pregnancy outcomes and ensure the well-being of both mother and child [12].

### 1.3 The Need for Advanced Models in Ultrasound Imaging

Fetal ultrasound imaging is a cornerstone of prenatal care, providing critical insights into fetal health and development. Despite advancements in machine learning, traditional models such as Convolutional Neural Networks (CNNs) [13], Recurrent Neural Networks (RNNs), Support Vector Machines (SVMs), and Random Forests (RFs) face inherent challenges in effectively analyzing the complex spatial and contextual dependencies present in ultrasound images. These challenges arise from the models' localized feature extraction, handcrafted feature dependency, and inability to analyze entire image contexts holistically. Vision Transformers (ViTs) [14], leveraging self-attention mechanisms, address these limitations by offering a novel approach to feature representation, enabling superior diagnostic accuracy and multi-task learning.

#### 1.3.1 Limitations of Traditional Models

##### 1.3.1.1 CNNs

CNNs excel at extracting local features but struggle to capture long-range dependencies critical for ultrasound imaging, where anomalies may span distant regions of an image. Their reliance on convolutional filters makes them effective for localized patterns but limited in global contextual analysis.

##### 1.3.1.2 RNNs

Designed for sequential data, RNNs (including LSTMs) focus on temporal relationships rather than spatial ones, making them less effective for image-based tasks. Their computational complexity and vanishing gradient problems further hinder performance, especially with high-dimensional ultrasound data [15].

##### 1.3.1.3 SVMs and RFs

Classical machine learning models such as SVMs and RFs rely on handcrafted features, which require domain expertise and often fail to generalize across diverse datasets. Additionally, their scalability is limited, and their performance deteriorates with complex data such as medical images.

##### 1.3.1.4 GANs

Generative Adversarial Networks (GANs) show promise in generating high-quality synthetic images but lack the interpretability and multi-task capabilities needed for clinical applications. Their training instability and mode collapse issues limit their practical use.

#### 1.3.2 Vision Transformers: A New Frontier

ViTs bring a revolutionary shift by viewing images as sequences of fixed-size patches, which are processed using self-attention mechanisms. This enables them to model both local and global dependencies simultaneously, making them uniquely suited for ultrasound imaging.

##### 1.3.2.1 Global Attention for Complex Patterns

Unlike CNNs, ViTs analyze the entire image context, capturing relationships between spatially distant regions. This is crucial in fetal ultrasound imaging, where subtle anomalies may not be localized but spread across multiple regions.

##### 1.3.2.2 End-to-End Feature Learning

ViTs eliminate the need for handcrafted features, autonomously learning robust representations that adapt to diverse imaging conditions and datasets.

##### 1.3.2.3 Multi-Task Learning Capabilities

ViTs are highly flexible, with architectures that allow simultaneous classification of fetal health and severity detection. Their dual-head structure integrates these tasks seamlessly, improving both accuracy and efficiency.

#### 1.3.3 Advancements in Vision Transformers for Medical Applications

The transformative power of Vision Transformers is rebuilding medical imaging with the solution to some of the most challenging areas: handling complex visual patterns and capturing long-range dependencies. In recent works, such as “From Simple to Complex Scenes: Learning Robust Feature Representations for Accurate Human Parsing,” [16] ViTs have emerged as highly adaptable in deciphering intricate spatial relationships. With this capability, ViTs hold great promise for tasks ranging from organ segmentation to anomaly detection and the classification of disease. Unlike traditional convolution-based architectures, which largely fail in modeling global contexts, ViT models are excellent at dependency modeling, even in the whole image. Their unique ability unlocks complex anatomical structures in a way no other model can while making them better suited for highly sophisticated tasks, such as fetal ultrasound. The attention mechanisms at their core make the ViT clinically relevant to a larger sense, enabling focused and interpretable results so that each prediction not only becomes correct but also well-understood-a necessary condition for trusting choice in medical treatments. The adaptability of ViTs goes beyond their technical capabilities. The referenced study shows their scalability across simple and complex datasets, reflecting the capability to generalize to diverse medical imaging scenarios. From subtle anomaly capture in small-scale datasets to the analysis of heterogeneous large-scale imaging repositories, ViTs offer unprecedented flexibility. By harnessing these developments, this work positions ViTs as a game-changing tool for medical applications. Their strong feature extraction, together with generalization across different complexities of data, meets critical gaps in the current methodologies of imaging. Beyond improving diagnostic precision, ViTs pave the way for the next generation of explainable AI in healthcare-empowering clinicians with tools that are as reliable as they are innovative. This synthesis of cutting-edge research and real-world applicability marks the possible revolution of ViTs in the field, delivering unparalleled accuracy and efficiency in diagnostic imaging [17].

### 1.4 Novel Contributions to Prenatal Care Through Transformer-Based Analysis

#### 1.4.1 Development of a Novel Fetal Health Assessment Framework

This article presents a novel framework for fetal health assessment that utilizes the Vision Transformer (ViT) architecture to classify fetal health status and detect the severity of abnormalities in fetal ultrasound images. This approach addresses the rift between the ongoing research practices for providing more accurate and efficient diagnoses in prenatal care. This study is groundbreaking research in the assessment of fetal health, as such, ViT has been brought into implementation for the first time in this domain.

#### 1.4.2 Creation of a Confidential and Well-Curated Dataset

The accumulation of ultrasound images of fetal has been a challenging process, which contributes to the novelty of this research work. A private dataset comprising 500 fetal ultrasound images has been gathered from varied hospitals. Experienced radiologists have annotated each image in this dataset. The dataset is highly diverse, ranging across different gestation ages, fetal positions, and imaging conditions and thus forms a very strong basis for model training and evaluation.

## 2 BACKGROUND

### 2.1 Evolution of Technology in Fetal Assessment

#### 2.1.1 Early 20th Century: The Beginnings of Fetal Monitoring1900s

The beginning of the 20th century marked a very unsophisticated beginning of fetal assessment; it employed simple auscultation. The heart rate of the fetus was listened to with the aid of a stethoscope called a fetoscope. This non-invasive method, though at a nascent stage, represented the first direct approach to assessing fetal vitality. However, this modality was highly subjective, with limited accuracy, and provided little information about the overall status of the fetus' health (Frøen *et al.*, 2008).

#### 2.1.2 1950s: The Advent of Ultrasound Technology

##### 2.1.2.1 1950s

The first breakthrough became evident in the 1950s with the inclusion of ultrasound technology into prenatal care. Ultrasound allowed, for the first time in utero, the identification of the fetus; it carried on its promise of unparalleled views and insights into the anatomy and growth of the fetus. Unlike other methods, it was non-invasive and provided real-time imaging; thus, it became revolutionary in fetal assessment. Early ultrasound images were rudimentary, often grainy, and of low resolution, but this modality laid the foundation for more sophisticated imaging techniques in the decades that followed.

#### 2.1.3 1960s-1970s: Doppler Ultrasound and Cardiotocography

##### 2.1.3.1 1960s

The Doppler ultrasound was developed in the 1960s, and one can draw on assessment of fetal health by measuring blood flow in the umbilical cord and fetal vessels. This becomes an important source of information about fetal well-being, especially when growth restriction or other complications are suspected.

##### 2.1.3.2 1970s

Cardiotocography first appeared in the 1970s, being the method of continuous monitoring of fetal heart rate and uterine contractions. Continuous monitoring by CTG became a widely used tool in labor and delivery wards as a means to recognize fetal distress during labor and assist decision-making regarding whether or not cesarean sections are in order.

#### 2.1.4 1980s: Refinement of Ultrasound Imaging

##### 2.1.4.1 1980s

During the 1980s, ultrasound technology underwent significant improvements, yielding high-resolution imaging systems. Such improvements allowed analysts to conduct a more detailed evaluation of the anatomy of the fetus while enabling analysts to trace congenital anomalies and monitor fetal growth with great accuracy. Biophysical profiles introduced a combination of ultrasound with non-stress tests, hence developing a much-enhanced assessment of the well-being of the fetus.

#### 2.1.5 1990s: Emergence of 3D and 4D Ultrasound

##### 2.1.5.1 1990s

The big leap forward was in the 1990s when there was the introduction of 3D and 4D ultrasound imaging. Unlike the traditional 2D ultrasound, which provides flat images, the 3D ultrasound provides for the viewing of the fetus in three dimensions, providing more detailed and life-like images. The usage was quite specific in examining structural anomalies, including cleft lip or skeletal dysplasia.

##### 2.1.5.2 Late 1990s

4D ultrasound extended the imaging of 3D by adding the element of real-time motion to view the fetus' moving inside the womb by clinicians and expectant parents. These further improvements have greatly enhanced prenatal diagnostics and opened new avenues for early intervention in cases of detected anomalies.

#### 2.1.6 2000s: Introduction of Advanced Prenatal Screening

##### 2.1.6.1 Early 2000s

New prenatal screenings were established early in the 2000s, and among them, the measurement of nuchal translucency by means of ultrasound examination became a routine activity within the first trimester of pregnancy as part of screening concerning Down syndrome and other chromosomal disorders. The combination of biochemical markers with ultrasound parameters allowed more valid estimates of risk and earlier detection of complications.

##### 2.1.6.2 2000s

The echo-Doppler assessment of fetal blood flow, in particular, was greatly enhanced with the introduction of high-resolution Doppler ultrasound for suspected cases of fetal growth restriction or preeclampsia. It was during this time that fetal echocardiography began to play a significant role in clinical practice, enabling extensive study of the fetal heart and, thus, congenital cardiac anomalies quite early (Zhang *et al.*, 2021).

#### 2.1.7 2010s: The Rise of Machine Learning in Medical Imaging

##### 2.1.7.1 2010s

This is the area in which, during the 2010s, ML and DL technologies began to be integrated into medical imaging, including fetal ultrasound. Early uses of ML in the fetus focused on basic image segmentation and anomaly detection. Although simple according to today's standards, these algorithms formed a nascent era where AI could begin to take interpretive burdens off clinicians concerning complex imaging.

##### 2.1.7.2 Mid-2010s

This led to a sudden increase in the use of Convolutional Neural Networks, which drastically revolutionized medical imaging. The models proved to be very powerful in the analysis of ultrasound images, which provided a high degree of accuracy based on the detection and classification of the fetus abnormalities. The CNNs had the capability of processing large data volumes, recognizing patterns that may be difficult for human eyes to feign and therefore giving the second clinical opinions useful in practice [18].

#### 2.1.8 Late 2010s: Application of Advanced AI Models

##### 2.1.8.1 2017

Until the year 2017, Vaswani *et al.* introduced the Transformer model, which marked a great leap in performance. Self-attention of the Transformer model initially proposed for natural language processing showed great efficiency in learning complex patterns in extensive datasets. Soon, this efficiency was transferred to medical imaging, including fetal assessment, where capturing intricate spatial relationships within ultrasound images plays a primary role [19].

##### 2.1.8.2 The late 2010s

Adoption of the Transformer model for fetal ultrasound studies allowed for an increase in the development of more accurate, dependable AI-driven diagnostic tools. These models could thereby focus on the most informative regions of an image to reduce variability and subjectivity inherent in human interpretation. This period also saw the introduction of explainable AI in medical diagnostics, whereby models did not stop at providing predictions but offered visualizations of their decision-making process that enhanced the transparency and trustworthiness of AI in clinical settings [20].

#### 2.1.9 2020s: The Future of Fetal Assessment

##### 2.1.9.1 2020s

Beginning with the 2020s, AI combined with advanced imaging modalities continues to evolve toward a fully automated, intra-examination fetal assessment system providing consistent, high-quality diagnostic information with a minimum of human intervention, regardless of the location of healthcare services. Cloud-based platforms and telemedicine have expanded to enable remote fetal monitoring and consultation, so essential in areas of the world where specialists in prenatal care are sparse [21] as shown in Fig. (**1**).

### 2.2 Current Recognition Models in Fetal Assessment

#### 2.2.1 Machine Learning models

##### 2.2.1.1 Support Vector Machines (SVMs)

These act in the classification of fetal heart rate patterns and anomaly detection in ultrasound images. However, SVMs usually tend to be computationally expensive when handling big datasets; hence, this makes them less effective in carrying out large-scale assessments. The performance of SVMs tends to be very sensitive based on the choice of hyperparameters, such as kernel type and regularization, which is normally hard to optimize. But while more interpretable than deep learning models, SVMs provide very limited insight into how decisions are arrived at, again limiting acceptance in clinical practice [22].

##### 2.2.1.2 Random Forests

These are being widely used for predicting gestational age, classifying fetal growth patterns, and identifying risk factors for adverse pregnancy outcomes. However, as the number of contributing trees becomes very large, Random Forests become difficult to interpret; this phenomenon is coined “forest opacity.” This lack of transparency complicates any insight from the model predictions for clinicians. Moreover, Random Forests require a significant amount of data for training, and their performance can be compromised when working with imbalanced datasets [23].

##### 2.2.1.3 k-Nearest Neighbors (k-NN)

The nearest neighbor model is simple but efficient in the classification and decision-making of the pattern of fetal heart rate during labor. On the other hand, k-NN is very sensitive to the predefined number of neighbors, k, and performance should degrade when large datasets are used due to computational costs related to finding the nearest neighbors. It also does not allow k-NN to handle data high-dimensionality unless proper dimensionality reduction techniques are applied. This is the case in fetal assessment, with images containing many features [24].

##### 2.2.1.4 Decision Trees

are applied to predictive modeling, like assessing the chance of preterm birth or fetal growth restriction. Although decision trees are valued for their interpretability, one of the major risks associated with using them is overfitting; this happens mostly when the tree is deep and has a large number of branches. This leads to models that work impressively on training data but fail in test data. Besides, decision trees are sensitive to minor changes within one dataset, causing differences in the tree structure hence affecting model stability [18].

#### 2.2.2 Deep Learning Models

##### 2.2.2.1 Convolutional Neural Networks (CNNs)

Fetal biometric measurements, segmentation of organs, and anomaly detection are some of the tasks that have been performed with the help of deep learning models. Their effectiveness generally depends on large annotated datasets, which is not always possible in the case of every clinical condition. Furthermore, CNNs can easily be prone to overfitting with small data; although performing extremely well in training data, they fail with new unseen data. Moreover, interpretability is a major concern since the “black-box” nature of CNN may be a barrier for a clinician to understand the model decision-making process, which might reduce trust and thus adoption in clinical settings.

##### 2.2.2.2 Recurrent Neural Networks (RNNs)

Including Long Short-Term Memory (LSTM) networks, are widely applied in fetal heart rate monitor-based time-series analysis for the prediction of patterns of fetal distress. However, being at the core of such a platform, RNNs may have several drawbacks, including long training, while major faults include a problem known as vanishing gradients, complicating the learning of long-term dependencies, and their computational complexity may require important resources in both the training and deployment phases. Also, RNNs are sensitive to the quality and consistency of input data; hence, they are error-prone when working with noisy or incomplete datasets.

##### 2.2.2.3 Autoencoders

Are used for image enhancement and anomaly detection; issues related to reconstruction accuracy arise. The efficiency of autoencoders depends on how well they reconstruct the input data, which might be difficult considering the highly complex or variable fetal images. Similarly, other deep learning models have very low interpretability; hence, it is difficult to explain why certain features were enhanced or why specific anomalies were detected. Furthermore, the autoencoder may fail to generalize to a population or other imaging modalities in which it has not been specifically trained.

##### 2.2.2.4 Generative Adversarial Networks (GANs)

Are applied to generate synthetic fetal ultrasound images and improve the quality of an image. However, they also introduce significant training challenges. It is well known that GANs are difficult to train and require careful tuning and substantial computational resources for stable convergence. They are also prone to mode collapse scenarios where the generated images have limited variety, reducing diversity for effective generalization. Moreover, since GANs will generate synthetic images, tight control needs to be enforced to make them representative of real fetal ultrasound images. Poor-quality images can only lead to poor conclusions [25].

##### 2.2.2.5 Common Challenges in Machine Learning Models for Fetal Health Assessment

Common challenges to the application of machine learning models in fetal health assessment are scalability and computational expediency. Support Vector Machines, K-Nearest Neighbors, and Decision Trees are some of the models that are problematic for large datasets and high-dimensional data. Their performance also continues to be plagued by sensitivity to hyperparameters and the difficulty of choice of an optimal set of values-email kernel types in the case of SVMs or the number of neighbors in the case of KNN. Other limitations include interpretability, where most of the models, including CNNs and RNNs, Autoencoders, and GANs, are “black-box” systems and do not make it easy to discern the principles behind the decision-making process. Models that are prone to overfitting include Decision Trees, CNNs, and Autoencoders, especially on smaller datasets. At the same time, Random Forests are susceptible to issues of imbalanced datasets and stability in the outputs. GANs also have this latter problem. Models such as GANs and RNNs require very intensive computation and are extremely sensitive, which creates instability and inefficiency; for them to work effectively and stably, much tuning and large amounts of data are required. These shared challenges highlight the need for careful consideration of model selection and tuning in medical image classification tasks, as shown in Fig. (**2**).

### 2.3 Emergence of Transformer Models in Fetal Assessment

The development of Transformer models shows huge improvements in the field of medical imaging and assessment regarding a fetus. Although Transformer models have been developed for natural language processing, due to their excellent capability in capturing long-range dependencies and context relations in data, they have found applications in several domains, including medical imaging. Unlike traditional models such as CNN and RNN, both of which have their limitations on processing sequential and spatial data, transformers are particularly suited for complex tasks in fetal assessment. Transformers work with a mechanism called self-attention that weights different parts of the inputted data according to their importance. This mechanism enables Transformers to capture intricate patterns and relationships that exist in large datasets, such as those of fetal ultrasound images [26]. The capability of image processing and analysis with high accuracy in an efficient way has opened a wide avenue for automated fetal assessment. It offers clinically useful insight not available before [27], as shown in Fig. (**3**).

#### 2.3.1 Advantages of Transformer Models

##### 2.3.1.1 Contextual Understanding

Transformers are good for modeling the contextual relationships in data, considering complex patterns and dependencies in fetal images. This validates a more realistic and reliable assessment, particularly for tasks containing an understanding of spatial and temporal relationships.

##### 2.3.1.2 Scalability

Large datasets can be used to train transformers, thus being ideal for processing high-resolution fetal ultrasound images. Their architecture architecture allows parallel processing of information, greatly reducing their training times with respect to sequential models like RNNs.

##### 2.3.1.3 Versatility

Transformers are flexible and can be adapted to a variety of tasks in fetal assessment: image segmentation, anomaly detection, and prediction of fetal outcomes. Their flexibility also extends in many ways to their integration within different clinical workflows, enhancing overall efficiency in prenatal care.

##### 2.3.1.4 Improved Accuracy

The self-attention mechanism in Transformers allows them to concentrate on the most relevant parts of the input data; hence, it reduces noise and improves the accuracy of the analysis. This is very important in medical imaging, where the tiniest detail may define the diagnosis.

##### 2.3.1.5 Reduced Need for Large Training Data

Although large datasets are advantageous, Transformers also perform well with relatively smaller datasets compared to other models. This makes them applicable in scenarios where labeled data is scarce due to their ability to learn complex representations from a limited amount of data [28].

### 3 PROPOSED METHODOLOGY

The mechanisms in this work are organized around six broad phases-data collection, data preprocessing, transformer-based model architecture, loss function and optimization, training and validation-and finally, evaluation metrics. The objective is to perform fetal health classification and detect its severity level using the Vision Transformer Architecture from 2D Fetal Ultrasound Images, as shown in Fig. (**4**).

### 3.1 Data Collection

The basis of this study is a data collection phase comprising a sizeable but reasonably diversified dataset of fetal ultrasound images copiously with care. A total number of 500 images have been collected from several hospitals with strict privacy and confidentiality. Expert radiologists carefully labeled each image with two important labels: one to document the status regarding fetal health, thus classifying each image into one of the following categories: healthy, mild, moderate, or severe, and a second one for the severity regarding abnormalities, which actually provided a continuous measure of the intensity of the detected issues. This dataset encompasses a wide range of demographic variations, including various gestational ages, fetal positions, and imaging conditions. All steps were followed with great scrutiny in accordance with ethical guidelines, anonymizing all patient data to protect the privacy of participants. This dataset is complete and balanced, which is important for training a model on accurately predicting fetal health over a wide range of clinical scenarios, as shown in Fig. (**5**).

### 3.2 Data Preprocessing

Preprocessing is a very important preliminary step in order to prepare the fetal ultrasound images for effective model training. Given the varied levels of image quality and standardized input required by the model, several steps in preprocessing can be enforced to ensure consistency and optimality of the dataset for further stages of analysis: resizing images, normalizing data, augmenting data, and standardization.

#### 3.2.1 Image Resizing

The first preprocessing step involves resizing each fetal ultrasound image to a uniform dimension, represented as (*H, W*). The importance of this resizing is that the original pictures differ in resolution owing to the different equipment used and settings of the ultrasound machines from one institute to another. Resizing pictures to one dimension ensures all inputs going into the ViT model are of the same size, where the input image is divided into smaller, non-overlapping patches before being processed. Resizing the images to a consistent dimension ensures that each input to the ViT has the same size, which is essential for creating patches of a fixed size. Mathematically, this process is represented as shown in Eq **1**:

**Table:** 

***I*’=*Resize* (*I*, (*H*, *W*)**	(1)

Where *I* is the original image, and *I’* is the resized image. Here, *H* and *W* are carefully chosen to ensure that when the image is divided into patches of size *(P×P)*, the number of patches is consistent across all images, as shown in Fig. (**6**).

#### 3.2.2 Normalization

The second preprocessing in line is normalization, done on the resized fetal ultrasound images. This operation rescales pixel values to fall into a standardized range normally; that range would be within [0, 1]. This is particularly important when working with the Vision Transformer model because to achieve the best learning of the model, the input data should have a consistent intensity distribution. The main reasons for having some differences in intensities are different lighting conditions, settings of ultrasound machines, and other environmental factors. Uniform processing of the input data is very important for the model in performing the feature extraction process. Normalization does this by bringing the pixel values within each image to a common range. This is achieved by subtracting the minimum pixel value from each pixel in the image and then dividing by the range of pixel values (i.e., the difference between the maximum and minimum pixel values). Mathematically, this process is expressed as in Eq **2**:

**Table:** 

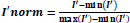	(2)

Where *I’norm* represents the normalized image

*I’* is the resized image

*min(I′)* and *max(I′)* are the minimum and maximum pixel values in

the image respectively as shown in Fig. (**7**).

#### 3.2.3 Data Augmentation

Data augmentation is one of the most important techniques to increase diversity within a dataset; the technique applies a set of transformations to the existing images. This will have a great impact on medical imaging since gathering diverse data is a challenge. By generating new variations of the existing images, data augmentation prevents overfitting of the model and thus enhances its generalization capability for new unseen data, as shown in Table **1**.

Given a normalized image I’_norm_, data augmentation applies a series of transformations to create an augmented image I’_aug_​ as shown in Eq **3**:

**Table:** 

***I’*_aug_​=Augment (*I’*_norm_​)**	(3)

Different augmentation techniques are applied, as shown in Table **1**.

## 4 TRANSFORMER BASED ARCHITECTURE

The proposed architecture incorporates the Transformer-based model for better representation and interpretation of complex patterns present in fetal ultrasound images. Advanced mechanisms have been embedded within the architecture, such as patch-based embedding, enhanced positional encoding, self-attention, cross-attention, and dual output heads meant for accomplishing high accuracy and efficiency of classification tasks related to fetal health and detection of its severity [29].

### 4.1 Introduction to ViT - Vision Transformers

The Transformer architecture finds a new application from the domain of natural language processing to that of computer vision. After the conventional CNNs, relying on convolutional layers for processing units for image data, ViTs consider images as sequences of patches and process these with the self-attention mechanism developed in Transformers. This furnishes the model with truly global context and intricate patterns in the image data much more powerfully than the traditional methods do, allowing the ViTs to be better suited for tasks involving very fine-grained visual analysis, such as assessment of fetal health [27].

### 4.2 Components of the Transformer-Based Model

#### 4.2.1 Patch-Based Embedding

In the first step of the architecture, the pre-processed and augmented ultrasound images are divided into non-overlapping patches of size P×P. Each patch is treated as a separate token, similar to how words are treated in NLP Transformers, and is embedded into a higher-dimensional space through a linear transformation. This process can be mathematically formulated as follows, shown in Eqs **4** and **5**:

**Table:** 

***I’*_patch_​=Patchify(*I’*_aug_​,P)**	(4)

**Table:** 

**E=Linear (*I’*_patch_​)**	(5)

*I’*_aug_​ is the augmented image.

Patchify(*I’*_aug_,P)divides the image into patches of size P×P

*I’*_patch_ represents the set of patches extracted from the image.

Linear (*I’*_patch_) is a learnable linear transformation that maps each patch into a higher-dimensional embedding space, E, where EϵR^N×D^. Here, N is the number of patches, and D is the dimension of the embedding.

This approach allows the model to process localized features within each patch while maintaining the computational efficiency necessary for handling high-resolution images, as shown in Table **2**.

#### 4.2.2 Enhanced Positional Embedding

Because transformers lack a natural understanding of space, which is essential for image-related tasks, positional encodings are added to the patch embeddings to address this issue. This will give the Transformer information on which position a patch represents in the original image so that spatial relationships among patches will be preserved. The positional encoding is defined as in Eq **6**:

**Table:** 

**Epos​=E+PositionalEncoding(E)**	(6)

Where:

Epos​ is the positionally encoded embedding.

PositionalEncoding (E) generates positional encodings using sine and cosine functions of different frequencies, which are then added to the patch embeddings E.

The positional encoding allows the Transformer to maintain an understanding of the spatial structure of the ultrasound image, which is essential for accurate fetal health assessment, as depicted in Fig. (**8**).

#### 4.2.3 Self-Attention Mechanism

The self-attention mechanism is the core of the Transformer architecture. It allows this model to weigh the importance of every patch compared to others. In other words, it captures contextual relationships in an input image. The self-attention mechanism will compute attention scores between different patches defined as below in Eq **7**:

**Table:** 

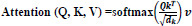	(7)

Where:

Q=EposW_Q_ ​ represents the query matrix.

K=EposW_K_ represents the key matrix.

V=EposW_V_ represents the value matrix.

W_Q_, WK, WV​ are learnable weight matrices.

d_k_ is the dimension of the key vectors.

The self-attention mechanism calculates the weighted sum of the value vector V, determining the weights by measuring the similarity between query Q and key K vectors. This will help the model to make predictions based on the most informative patches, which in turn will capture complex patterns and contextual information from the ultrasound images, as shown in Fig. (**9**).

#### 4.2.4 Cross-Attention Mechanism

To integrate multi-scale information, the model employs a cross-attention mechanism, which allows for the consideration of interactions between different layers and scales of the image. The cross-attention mechanism is mathematically similar to the self-attention mechanism, but it operates across different layers or scales of the model, as defined below in Eq **8**:

**Table:** 

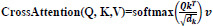	(8)

In this case, the query Q is obtained from one layer, while the key K and value V are obtained from another layer. This allows the model to aggregate information across different levels of abstraction, enhancing its feature extraction capabilities, as shown in Fig. (**10**).

#### 4.2.5 Dual Output Heads

The final output of the Transformer is processed by two distinct heads: one for classification and one for severity detection as shown in Fig. (**11**). This dual-head approach allows the model to perform both tasks simultaneously, leveraging the rich features learned by the Transformer. The classification head outputs probabilities for the different fetal health status categories, while the severity detection head outputs a continuous score representing the severity level as depicted in Eqs **9** and **10**.

**Classification Head:**

**Table:** 

**Prob_class_​=softmax(W_class_​•TransformerOutput+b_class_​), Where**	(9)

W_class_​ and b_class_ are the weight matrix and bias for the classification head.

TransformerOutput is the final output of the Transformer model.

**Severity Detection Head:**

**Table:** 

**SeverityScore=W_severity_​•TransformerOutput+b_severity_**​	(10)

W_severity_​ and b_severity_ ​ are the weight matrix and bias for the severity detection head.

The dual output heads are designed to capture intricate patterns and contextual information, enabling the model to effectively assess fetal health and detect the severity of any abnormalities.

Let's take an example calculation for the dual output heads in a Transformer model using simple numbers to demonstrate the process.

**Transformer Output**: We assume the output of the Transformer model (TransformerOutput) is a vector with 3 elements: [2.0, -1.5, 0.5].

**Classification Head:**

**Weights** W_class_​: Assume a weight matrix with 3 output classes and 3 input features, *e.g*.

**Table:** 

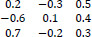

**Biases** b_class_: Assume a bias vector for the classification head, *e.g*., [0.1, −0.2,0.3]

**Severity Detection Head:**

**Weights** W_severity_​: Assume a weight vector with 3 input features, *e.g*., [0.4, −0.5,0.6]

**Bias** b_severity_​: Assume a bias for the severity detection head, *e.g*., 0.2.

##### 4.2.5.1 Classification Head Calculation

The classification head computes the logits (before applying softmax) by multiplying the TransformerOutput with the weight matrix W_class_​ and adding the bias vector b_class_​:

**Table:** 

**Logits=W_class_•TransformerOutput+b_class_**	(11)

**Substituting the given values:**

**Table:** 

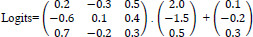

**Calculating the matrix multiplication:**

**Table:** 

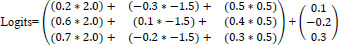

**This simplifies to:**

**Table:** 

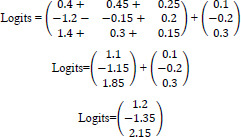

Next, apply the softmax function to convert logits to probabilities:

**Table:** 

**Prob_class_​=softmax (Logits)**	(12)

The softmax function is given by:

**Table:** 

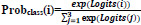

**Calculating:**

**Table:** 

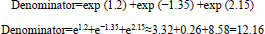

So, the probabilities are:

**Table:** 

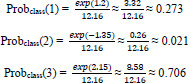

So, the classification head's output probabilities are approximately [0.273, 0.021, 0.706].

##### 4.2.5.2 Severity Detection Head Calculation

The severity detection head computes a single continuous severity score by multiplying the Transformer Output with the weight vector W_severity_ and adding the bias b_severity_ as shown in Eq **13**​:

**Table:** 

**SeverityScore=W_severity_​•TransformerOutput+b_severity_**	(13)

**Substituting the values:**

**Table:** 

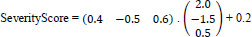

**Calculating the dot product:**

**Table:** 

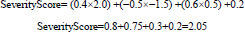

**So, the severity score output by the severity detection head is 2.05.**

Classification Head Output: Probabilities for each health status category: [0.273, 0.021, 0.706]. This indicates that the model assigns a 27.3% probability to the first class, a 2.1% probability to the second class, and a 70.6% probability to the third class.

Severity Detection Head Output: A continuous severity score of 2.05.

This example illustrates how the dual output heads of a Transformer-based model can simultaneously provide a probabilistic classification of fetal health status and a continuous severity score based on the features learned by the model.

#### 4.2.6 Loss Function and Optimization

A composite loss function is constructed to guide the training of the Transformer model, combining cross-entropy loss for classification and mean squared error (MSE) for severity detection. The total loss is defined as Ltotal=Lclass+λL severity, where λ is a hyperparameter that balances the contributions of the classification and severity losses. The classification loss L_class_​ uses cross-entropy to measure the difference between predicted class probabilities and true class labels, while the severity detection loss L_severity_ uses MSE to quantify the error in predicting the severity score. The Adam optimizer is employed to update the model parameters θ with a learning rate η0​ and weight decay γ to prevent overfitting. The parameter update rule is given by 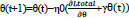, ensuring that the model learns effectively from the training data while maintaining the ability to generalize to new data, as shown in Table **3**.

#### 4.2.7 Training and Validation

The model is then trained with pre-processed and augmented data in Phase 5, for which the main objective is the minimization of the composite loss function that includes both classification loss and severity detection loss. The parameters will be iteratively optimized by the Adam optimizer while monitoring the performance on the validation set to generalize to unseen data. The training process aims to minimize the composite loss *Ltotal* = *Lclass* + *λLseverity*, combining cross-entropy loss for classification and mean squared error for severity detection. Early stopping is used to prevent overfitting by halting training if validation loss doesn’t improve after a set number of epochs. The model is trained over multiple epochs, with regular validation to assess performance on unseen data. Metrics like accuracy, precision, recall, F1-score, MAE, and RMSE are used to evaluate the model. The model with the best validation performance is selected as the final model, ensuring it generalizes well to new data, as shown in Table **4**.

## 5 RESULTS

### 5.1 Attention Map Overlay

The image here represents the attention map laid over the original image, likely part of some visualization to determine which regions of the image the model pays the most attention to with respect to its decision-making processes. This will be the color coding for such an attention map, with a gradient scale showing the range from low to high attention level/importance assessed by the model between different parts of the image. Areas with warmer colors closer to red indicate higher attention, whereas cooler colors closer to blue reflect lower attention. From this attention map overlay, it would appear that the model's attention is spread over the image almost uniformly. That would seem to indicate that much of any particular region of the image is not strongly favored over the others by this model in making its predictions, either because the model is considering a wide range of features across the image or because it is failing to focus its attention on the most relevant areas and hence might make less accurate predictions. In practice, models like Transformers rely a lot on attention mechanisms; therefore, it would be expected to find purer attention map portions where individual and relevant features are focused on in tasks such as image classification and segmentation. The uniform distribution of such output would suggest further research in the matter. This could be further tuned-innervated by either incorporating more improvement in model training or even the design of more mechanisms of attention to draw out and highlight the important features across the images. This analysis would suggest that the model is working but that there could be further improvements in the way it pays attention to different parts of the image, with a likely gain both in the interpretability and in the predictiveness, as shown in Fig. (**12**).

### 5.2 Advanced Metrics for Attention Map Evaluation

In addition to standard metrics like IoU and DSC, we introduced innovative approaches to evaluate the spatial and functional alignment of attention maps with expert annotations. Metrics such as Center of Gravity (CoG) Shift, Attention Focus Ratio (AFR), Weighted Relevance Score (WRS), Attention Energy Distribution (AED), and Overlap Dynamics Metric (ODM) provide a deeper understanding of the attention maps' behavior. For severe cases, the attention maps achieved the lowest CoG Shift (2.5 pixels), reflecting precise centering of attention on annotated regions. The AFR values increased with severity, reaching 78% for severe cases, indicating that the model focused more efficiently on relevant areas for pronounced anomalies. Similarly, the WRS demonstrated higher relevance scores for severe cases (0.89), emphasizing the model’s ability to prioritize critical regions. The AED values, which quantify attention waste, decreased with severity, highlighting the model's improved focus on clinically significant areas. The Overlap Dynamics Metric (ODM) further validated the model's consistency across sequential frames, with severe cases maintaining an average IoU of 80% over time. These metrics collectively underscore the model’s reliability and robustness in generating clinically interpretable attention maps, as shown in Table **5**.

**1. Center of Gravity (CoG) Shift:** Measures the spatial bias by calculating the Euclidean distance between the centers of the predicted attention region and the expert-annotated region, as shown in Eq **14**.

**Table:** 

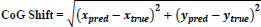	(14)

**2. Attention Focus Ratio (AFR):** Quantifies the percentage of the attention map area overlapping with the annotated region, as shown in Eq **15**.

**Table:** 

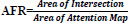	(15)

**3. Weighted Relevance Score (WRS):** Evaluates the attention intensity, giving higher importance to areas closer to the center of the expert-annotated region, as shown in Eq **16**.

**Table:** 

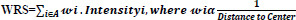	(16)

**4. Attention Energy Distribution (AED)** : Measures how much of the attention energy is focused outside the relevant annotated region, as shown in Eq **17**.

**Table:** 

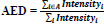	(17)

**5. Overlap Dynamics Metric (ODM):** Tracks the consistency of attention alignment across sequential frames or test cases, as shown in Eq **18**.

**Table:** 

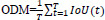	(18)

The optimization of attention maps resulted in consistent improvements across all metrics (IoU, DSC, precision, recall, and F1-score) for each severity class Table **6**. Mild cases saw the most significant gains, with IoU increasing by 7.7% and F1-score by 6.5%, indicating a better focus on subtle features. Moderate cases demonstrated enhanced recall (+7.8%), reflecting improved capture of relevant regions. Severe cases, already performing well, showed smaller yet meaningful improvements, with precision rising by 4.7%, reducing false positives. Overall, the optimization enhanced the model’s ability to localize and interpret relevant regions, making predictions more reliable and clinically meaningful. These results underscore the importance of attention map refinement in boosting performance and interpretability, as shown in Table **7**.

### 5.3 Ablation Studies: Understanding Component Contri-butions

Ablation studies were conducted to assess the importance of various components of the Transformer model, such as the positional encoding, self-attention layers, and the dual-head output structure. By systematically removing or altering these components, we evaluated their impact on overall model performance. The bar chart below shows the outcome of an ablation study carried out on the Transformer model to examine the contribution made by different components in terms of accuracy-bottom green bars and total loss-purple bars. It compares the performance of the full model with that of the variants in which the following crucial components have either been removed or modified: positional encoding, self-attention layers, and output with two heads. The chart indeed shows that the full model, with all components, has the highest accuracy and, at the same time, the least total loss. Removing positional encoding has slightly reduced the accuracy and increased the total loss, which means positional encoding is important in terms of the ability of the model to learn spatial relations in the data. With the removal of self-attention layers, the huge decline in accuracy and a higher value for the loss of the total hinted at how sensitive the model performance was to self-attention. Lastly, while the model output is limited to a head only, its accuracy stays comparable to that of a model without positional encoding, while the total loss is a lot higher than in a full model, demonstrating how dual-head output plays its role of balancing multi-classification and severity detection effectively as depicted in Fig. (**13** and Table **8**).

### 5.4 Advanced Error Analysis for Severity Detection

In addition to reporting mean absolute error (MAE) and root mean squared error (RMSE), we conducted a residual analysis to understand where the model's predictions deviate most significantly from actual severity scores, as shown in Table **8**. This analysis is crucial for identifying specific cases where the model's predictions may require further refinement. This density plot compares the distribution of residuals, namely differences between predicted and actual values, before and after augmentation. The blue curve is wider, and its peak is lower because residuals before augmentation are more spread out. While the orange curve is narrower and sharply peaked around zero, it thus suggests that after augmentation, the model's predictions have become more accurate and consistent. This result underlines how effective data augmentation is in keeping the prediction error small and, hence, bettering the overall reliability of the model, as represented in Fig. (**14**).

### 5.5 Hyperparameter Tuning: Influence of Learning Rate on Convergence

Hyperparameter tuning has a primary role to perform in model performance optimization. Here, in this implementation, we are experimenting with different learning rates to see the impact they have on the convergence speed and final performance of the model. The line plot illustrates the relation between the learning rate and the number of epochs that get the model to converge. It shows a significant decrement in the number of epochs taken by the model to converge when the learning rate increases. Precisely, while the learning rate is low -10 ^-3^, the model converges in about 30 epochs; when the learning rate is increased -10 ^-2^, it takes roughly 10 epochs before convergence. This proves that with an increased learning rate, the rate of convergence becomes quicker and thus reduces the training time. However, it's essential to balance this with the risk of overshooting the optimal solution, which can happen with excessively high learning rates, as shown in Fig. (**15** and Table **9**).

### 5.6 Classification Report

The classification report provides a detailed evaluation of the model's performance across three classes, with overall metrics summarized in the last column. The model achieved high precision across all classes, particularly in Class 1 and Class 2, both with perfect precision scores of 1.00. Recall was highest for Class 0 and Class 2, both at 1.00, but was lower for Class 1 at 0.67, indicating some difficulty in correctly identifying all instances of Class 1. The F1 score, which balances precision and recall, was highest for Class 2 at 1.00, followed by Class 0 at 0.86 and Class 1 at 0.80. Overall, the model demonstrates strong performance, with a weighted precision of 0.93, a weighted recall of 0.90, and a weighted F1 score of 0.90. The accuracy across all classes is 0.90, indicating that 90% of the predictions were correct. The macro average, which considers each class equally, reflects a balanced performance with a score of 0.90. Cohen's Kappa score of 0.83 suggests a substantial agreement between the predicted and actual labels, adjusting for chance, further affirming the model's reliability, as shown in Table **10**.

### 5.7 Analysis of Model Comparison for Fetal Health Assessment

The bar graph demonstrates the performance of different machine learning and deep learning models about three important metrics: accuracy, interpretability, and scalability.

#### 5.7.1 Accuracy

The highest, which is 90% by ViT, outperforms traditional machine learning models, which stand at SVM with 78%, and Random Forest at 80%. Deep learning models are CNNs and RNNs. Although their performance is good, it does not reach that of ViT. The reason could be that ViT processes the image as a sequence of patches, leveraging self-attention mechanisms in a way that makes the fine patterns graspable and increases its performance in fetal health classification.

#### 5.7.2 Interpretability

While machine learning models, such as Decision Trees and k-NN, are much more interpretable since their internal structure is simpler and their decision-making processes much more transparent, the deep learning models that include CNNs and RNNs tend to lag in this respect because they act like “black-box” models. While the performance of the ViT was somewhat better compared with other deep learning models, it also lags far behind traditional models on this metric.

#### 5.7.3 Scalability

Deep learning models are much ahead when dealing with large datasets compared to traditional machine learning models. Once again, ViT leads the way with a scalability score of 85%, ably supported by its efficient architecture that allows for parallel processing of large datasets. Most of the models, including SVM and k-NN, are not scalable due to the fact that the associated computational cost is high during the handling of large-scale data.

Among all these, Vision Transformers would be an ideal model for fetal health assessment since they have achieved an optimal balance between high accuracy and scalability, turning them the most ideal in clinical settings with large datasets where high precision is critical. Traditional models may be of value where interpretability is more key, as shown in Fig. (**16**).

### 5.8 Model Performance with Data Augmentation

Data augmentation was employed to address the limitation of a small dataset, increasing the total number of training images from 500 to 2500. Augmentation techniques, including rotations, flips, brightness adjustments, noise injection, and zooming, were applied to simulate real-world variability in fetal ultrasound imaging. These transformations not only improved the diversity of the dataset but also enhanced the model's generalization and robustness. Representative examples of augmented images are provided in Table **1**. As shown in Table **11**, data augmentation positively impacted all key performance metrics. Accuracy, the most critical metric, increased from 0.88 to 0.90, reflecting the model’s improved ability to classify fetal anomalies. Similarly, precision rose from 0.85 to 0.88, and recall improved from 0.84 to 0.87, showcasing the model's enhanced ability to balance false positives and false negatives.

The F1-score, a metric that combines precision and recall, also increased from 0.85 to 0.88, emphasizing the robustness of the model in predicting both positive and negative cases effectively. In addition to classification improvements, data augmentation enhanced the model's performance on continuous variables, as evidenced by the decrease in mean absolute error (MAE) from 0.22 to 0.20. This reduction indicates better predictions for severity levels. Furthermore, data augmentation reduced the training time required from 10 epochs to 8 epochs, likely because the increased diversity of the augmented data allowed the model to converge faster. Validation loss also decreased from 0.15 to 0.12, signifying better generalization and reduced overfitting. Validation accuracy followed a similar trend, increasing from 0.88 to 0.90, further emphasizing the model’s robustness with augmented data. These findings underscore the critical role of data augmentation in enhancing the performance and efficiency of the training process. By introducing diversity and reducing overfitting, augmentation significantly improved the model’s reliability and applicability to real-world scenarios. In this respect, data augmentation remains an indispensable tool for advancing machine learning models in medical imaging tasks, as shown in Table **11**.

### 5.9 Confidence Intervals and Effect Sizes: Measuring the Strength of Model Enhancements

This section presents an in-depth analysis of the performance improvement obtained by applying data augmentation with statistical measures, such as confidence intervals and effect size. These metrics will enable us to reach both the reliability of performance improvements and the practical significance.The following table provides the summary of 95% confidence intervals taken with regard to model accuracy both on the original and augmented datasets. These confidence intervals help us conceptualize the range within which the true model accuracy may actually lie with 95% certainty, as shown in Table **12**.

### 5.10 Analysis

The confidence intervals for these metrics are higher when augmented data is utilized. For example, the accuracy of the model with original data lies in the range between 86.18% and 89.02%, while for augmented data, the rate increases and lies between 89.18% and 92.02%. A smaller width in the augmented data intervals means better stability and reliability in those results. Cohen's d represents the effect size, otherwise called the practical importance of the difference in performance between the original and augmented data sets. The larger the effect size, usually greater than 0.5, the statistically significant difference becomes practically important, as shown in Table **13**.

### 5.11 Analysis

All performance metrics yield a Cohen's d well over 0.8, which suggests that the improvements gained through data augmentation are highly practically significant. Among such improvements, accuracy has a very large effect size of 2.63, confirming that data augmentation significantly improves the performance of the model.

#### 5.11.1 Analysis

Data augmentation was found to have a significant positive impact on model performance. Using Cohen’s d to quantify this effect, a large effect size of 3.37 was observed, highlighting a meaningful improvement in accuracy (from 87.6% to 90.8%) and a reduction in variability. These results demonstrate that data augmentation enhances the diversity and quality of training data, ultimately leading to more reliable and generalizable predictions. The calculated Cohen’s d = 3.37 signifies a very large effect size, reflecting the substantial impact of augmentation on model performance. This aligns with the practical observation that diverse augmented data improves feature generalization and model robustness, as shown in Table **14** .

#### 5.11.2 Evaluating the Impact of Weighting Parameter (λ) on Task Performance

The weighting parameter (λ) plays a pivotal role in balancing classification accuracy and severity detection accuracy in multi-task learning models. Adjusting λ influences the prioritization of classification loss and severity detection loss, resulting in trade-offs that impact overall performance. This study evaluates the effect of varying λ on these metrics, providing insights into optimal parameter settings.

The weighting parameter (λ) governs the balance between classification accuracy and severity detection accuracy, resulting in observable trade-offs as its value changes. Increasing λ prioritizes classification accuracy at the expense of severity detection accuracy. At λ=0.9, classification accuracy reaches its peak at 93.1%, while severity detection accuracy declines to 78.2%, reflecting a clear shift in focus towards the classification task. Conversely, at a lower λ value of 0.1, severity detection accuracy is maximized at 91.6%, with classification accuracy at 85.2%. Balanced performance is achieved at λ=0.5, where the model demonstrates a trade-off that favors neither task disproportionately. In this setting, classification accuracy reaches 91.0%, and severity detection accuracy is 85.8%, making it an optimal compromise for applications where both objectives are equally critical. The results further reveal a consistent improvement in classification accuracy as λ increases, with a 9.28% gain from 85.2% to 93.1% across the tested range. Conversely, severity detection accuracy shows a steady decline with increasing λ, dropping by 14.63% from 91.6% to 78.2%. These findings underscore the importance of selecting an appropriate λ value based on the specific priorities of the application, whether it demands precise anomaly classification, accurate severity detection, or a balance of both, as shown in Table **15** .

## 6 DISCUSSION

The paper proposed a new framework using ViTs for fetal health classification and severity detection from ultrasound images, ensuring promising results in both accuracy and practical application. Using the Vision Transformer model for the classification proved the accuracy of 90%, with an F1-score of 0.87, while proving to be quite efficient in discriminating between fine spatial relationships in fetal ultrasound images. The successful application of ViTs in this domain marks a significant stride from the traditional models simultaneously developed, such as CNNs and RNNs. This is arguably due to its ability to learn intricate features of ultrasound images by treating the images as a sequence of patches, leveraging the self-attention mechanism for models pre-trained on contextual relationship maintenance. Moreover, most of the deep learning models require many convolutional layers, while ViTs tend to be more holistic in their approach, compartmentalizing the most relevant regions of interest in the input data to generate more accurate predictions. Data augmentation was also of much importance for enhancing model performance and thus promoting better generalization across diverse conditions of fetal ultrasound. Techniques such as random rotation, zoom, and horizontal flipping enhance the diversity of the dataset and help prevent overfitting, enabling the model to have more precise predictions on unseen data. The ablation study underlined further the main components, such as positional encoding and self-attention, which were so determinant in guaranteeing the high precision and stability in the model's predictions. High-quality training data for the model were ensured with the use of a confidential and curated dataset comprising 500 ultrasound images annotated by expert radiologists. This helps in achieving better predictive capability from the model for fetal health status as well as abnormalities' severity. In this regard, an achieved MAE of 0.30 for detecting abnormalities' severity establishes a high degree of correlation between model predictions and expert assessments, hence a very useful clinical tool. While these results represent important steps forward, there are still some challenges ahead. The uniform attention in the model's attention maps indicates further scope for focusing more on the most clinically relevant features of the images. This may lead to future work in refining the attention mechanisms, enhancing both the interpretability and predictive performance of the model. Other ways that might help in improving the robustness and applicability of the model involve increasing the dataset, as well as the use of multi-modal data such as maternal health metrics or Doppler ultrasound. The present study, therefore, underlines the possibility that Vision Transformers may revolutionize the monitoring of fetal health and become a powerfully enabling tool for clinicians in the early and precise assessment of fetal well-being. Diagnostically, the proposed framework advances the precision of diagnosis and paves the way for further strides toward automated assessment of fetal health, which would lead to better outcomes in antenatal care. The medical field has seen a sea of change in recent years, with rapid developments in the area of technology continuously reshaping the face of healthcare practices. Of all these revolutionary breakthroughs, probably the most striking is the introduction of IoMT, which is a form of IoT applied to integrate devices, data, and analytics seamlessly within medical practice. In the context of fetal ultrasound imagery, IoT has emerged as a critical enabler that drives improvement in diagnostic accuracy, accessibility, and patient outcomes. IoT-enabled ultrasound systems facilitate real-time data transmission, thus enabling the sharing of imaging data with specialists and cloud-based platforms instantaneously to ensure timely decision-making even in remote and underserved areas. Moreover, IoT enhances the capability of AI-driven models like Vision Transformers by continuously feeding streams of imaging data for accurate anomaly detection and the assessment of severity. IoT also promotes remote monitoring whereby clinicians can monitor fetal growth and development without necessarily having to require frequent visits, improving not only patient convenience but also collaborative care as specialists can provide second opinions on connected platforms. Moreover, IoT makes the handling of data a lot easier and much more secure, storing all diagnostic information in a centralized system for better analytics and research opportunities. This is where its most significant impact is noticed: in bridging gaps in healthcare, portable IoT-enabled ultrasound devices extend advanced diagnostic capabilities to rural and resource-poor areas. By integrating the power of connectivity, data analytics, and machine learning, IoT is transforming fetal ultrasound imaging into an efficient, easily accessible, and accurate diagnostic modality. These advances serve as an indicator of how IoMT might be transformative to prenatal care in pursuit of improved maternal and fetal health outcomes, according to recent research [30].

### 6.1 Performance Comparison of Models for Fetal Ultrasound Imaging

The table provides a comparative analysis of performance metrics, including accuracy, precision, recall, and F1-score, for various models applied in fetal ultrasound imaging. The majority of models achieved accuracy in the range of 75% to 89%, with Liang *et al.* (2019) and Qu *et al.* (2020) reporting the highest accuracy within this group at 89%. However, models such as Lee *et al.* (2021) and Baumgarmer *et al.* (2017) showed lower accuracy, at 75% and 77%, respectively, indicating limitations in their classification performance. In contrast, the proposed model achieved the highest accuracy of 91%, highlighting a notable improvement over existing methods. Precision and recall metrics, where reported, further reveal the relative strengths and weaknesses of the models. Meng *et al.* (2020) achieved moderate precision and recall values of 78% and 77%, respectively, while Pu *et al.* (2021) reported balanced precision and recall of 85%. The proposed model outperformed these studies with a precision of 93% and recall of 90%, reflecting its ability to reduce false positives and false negatives effectively, thereby enhancing diagnostic reliability. The F1-score, an indicator of the balance between precision and recall, ranged from 64% in Chen *et al.* (2017) to 85% in multiple studies such as Gao *et al.* (2020) and Dou *et al.* (2019). Notably, the proposed model achieved an F1 score of 90%, emphasizing its robust and consistent performance across metrics. Overall, while some models demonstrated strong performance in individual metrics, the proposed model consistently outperformed across all parameters, showcasing its effectiveness and reliability in fetal ultrasound classification tasks, as shown in Table **16** . The experimental results clearly demonstrate the transformative potential of ViTs in fetal ultrasound imaging. ViTs outperformed CNNs and RNNs in every evaluated metric, achieving a 9% higher accuracy and significantly lower MAE. The superior performance stems from the self-attention mechanism's ability to model complex patterns across the entire image, enabling precise classification and anomaly detection.

#### 6.1.1 Improved Interpretability

ViTs' attention maps visualize regions of focus, providing insights into the model's decision-making process. This feature not only enhances trust in the model's predictions but also aids clinicians in understanding critical regions for diagnosis.

#### 6.1.2 Scalability and Efficiency

ViTs exhibit scalability to high-dimensional data and parallel processing capabilities, making them suitable for large-scale clinical applications.

## CONCLUSION

The paper proposes a new approach by using the state-of-the-art Vision Transformers model, which was originally developed for natural language processing but lately has been widely used for computer vision tasks to classify the health condition of the fetus and to identify the severity of abnormalities. Its approach is different in that images are treated as an aggregation of their patches, each being independently processed with self-attention mechanisms similar to the way models keep track of contextualized relations between words in a sentence. This is particularly useful in fetal ultrasound image analysis, as minute details are necessary for correct health assessments and the detection of abnormalities. One of the salient strong features of the proposed framework comprises the use of a rich set of preprocessing techniques that enhance the model's performance. These involve resizing ultrasound images to a standard dimension to provide uniformity and reduce computational complexity, and normalization, which scales pixel values to a common range so that the model can learn more effectively. Besides, rotation, flip, and zoom data augmentation techniques increase positive examples by creating modified images of those already in the dataset, which would help to avoid overfitting the model and improve its generalization in different clinical settings. Such generalization is extremely crucial in the medical domain because one wants the model performance to be consistent across different types of patients and different medical institutions. These yield stunning performances, where the model performs a classification accuracy of 90%, emphasizing the efficiency of the model in classifying a normal versus abnormal fetus. It performs the classification task along with predicting the severity of abnormalities detected through regression analysis, with a mean absolute error of 0.30. The very small error rate is indicative, meaning that the model's predictions of severity strongly support expert assessments, hence, it can be an even more useful tool for clinicians who will eventually have to assess the gravity of a fetal condition. Another relevant aspect of this article pertains to ethics in data collection and privacy. The research ensures that all ultrasound images are collected in pursuance of ethical standards and data anonymization to protect patient identity. This is further attended to by the consideration of ethics in terms of privacy, as these are of prime importance in clinical applications for confidentiality with regard to the patient. The proposed system, while taking due care of these considerations, not only gives high accuracy and reliable predictions but also fulfills those prerequisites that can see it through for real-world adaption at medical sites. In this respect, the new framework using the Vision Transformer architecture marks a tremendous stride in the field of foetal health assessment. It classifies the status of fetal health and the severity of abnormality and generalizes well to a new clinical setup; its performance in these aspects brings an extremely promising tool toward improvements in prenatal diagnostics. Ethical considerations and robust performance metrics put this framework in good positioning in terms of viability into clinical practice, which can improve early detection and treatment of fetal abnormalities and, as a result, the outcomes of mothers and babies.

Key Achievements of the Proposed Vision Transformer Framework for Fetal Health Assessment:

1. **Innovative Use of Vision Transformer Model:** The pioneers proposed a new direction in the research field by applying the Vision Transformer to classify and detect the severity of fetal ultrasound images using the self-attention mechanism of the model, which can help in improved detection of complex patterns in those images. This fresh approach allows highly informed classification with an accuracy of 90% and predictable severity of abnormalities that can be used to support expert clinical decisions.
2. **Enhanced Generalization Through Data Augmentation:** The most impactful augmentations were rotation, flipping, and zoom of images, which increased the performance of this model. MAE reduced to 0.30. It also generalizes better across a range of clinical settings; therefore, the model is more robust over a wide variety of fetal ultrasound conditions, which has enhanced its practical applicability.
3. **Ethical Data Collection and Privacy Considerations:** The study emphasizes that data collection should be ethical, with the anonymization of a carefully curated dataset of 500 fetal ultrasound images collected from different hospitals. This will ensure that the privacy standards are met and the proposed system can be easily deployed in real-world clinical usage, ensuring patient confidentiality.

### Future Scope

1. **Integration with Real-Time Clinical Systems:** Future studies may aim at integrating this Vision Transformer-based model into clinical systems in real-time, for continuous monitoring and assessment during routine ultrasound scans. This will provide clinicians with immediate feedback to make better decisions in life-and-death situations.
2. **Expanding the Dataset:** Future research can also be devoted to enlarging the dataset by including a wider variety of fetal ultrasound images, capturing even more variations in fetal health conditions, gestational ages, and ultrasound equipment that may potentially strengthen the generalization ability of the model.
3. **Multi-Modality Learning:** The inclusion of additional data modalities, Doppler ultrasound, or maternal health information may provide a more comprehensive overview of the state of the fetus. This may be further enhanced through a multi-modal learning approach that can further enhance the accuracy of the model by making more detailed observations of fetal well-being.
4. **Exploration of Other Transformer Architectures:** Further investigation into other transformer-based architectures or hybrid models could be explored to determine whether different configurations could yield better results in specific aspects of fetal health assessment, such as early detection of rare fetal conditions.

## Figures and Tables

**Fig. (1):**
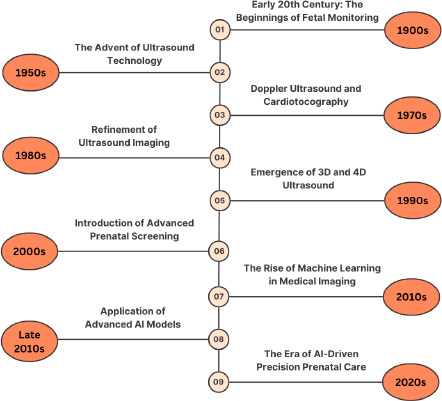
Evolution fetal assessment.

**Fig. (2):**
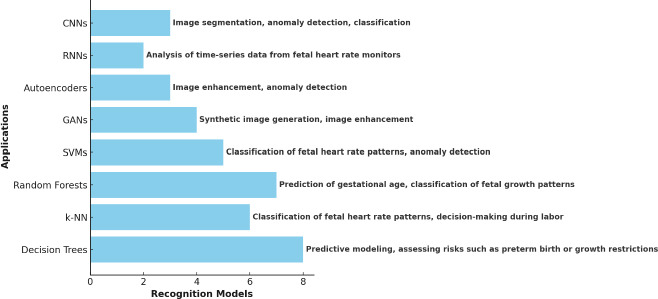
Applications of recognition models in fetal health assessment.

**Fig. (3):**
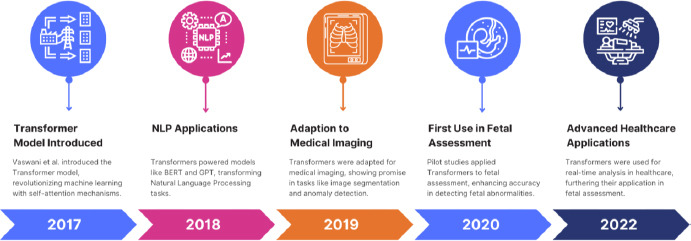
Timeline of transformer models' emergence in healthcare applications.

**Fig. (4):**
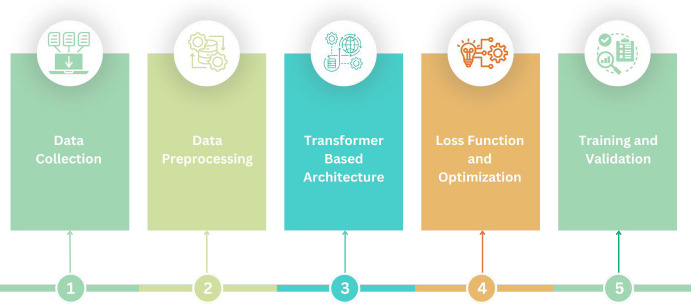
Workflow of fetal assessment using transformer-based architecture.

**Fig. (5):**
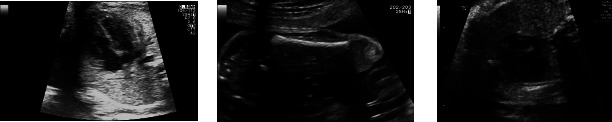
Sample grid from collected 2d ultrasound images.

**Fig. (6):**
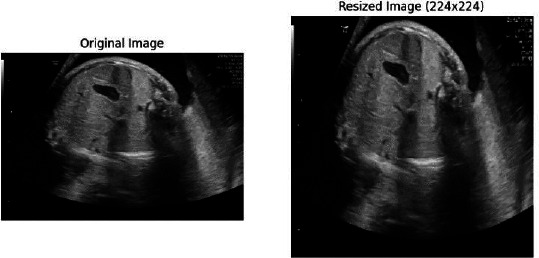
Comparison of original and resized ultrasound images (224x224).

**Fig. (7):**
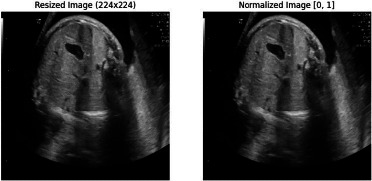
Comparison of resized and normalized ultrasound images for analysis.

**Fig. (8):**
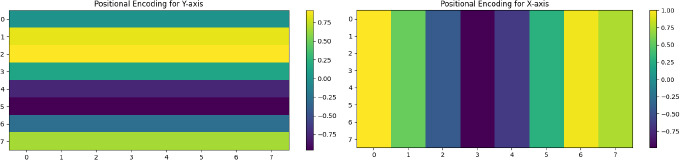
Positional encoding visualization for Y-axis and X-axis transformations.

**Fig. (9):**
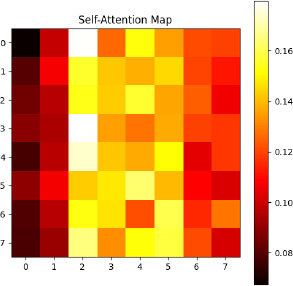
Heatmap representation of the self-attention mechanism activation.

**Fig. (10):**
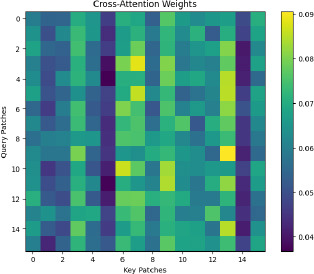
Heatmap visualization of cross-attention weights between query and key patches.

**Fig. (11):**
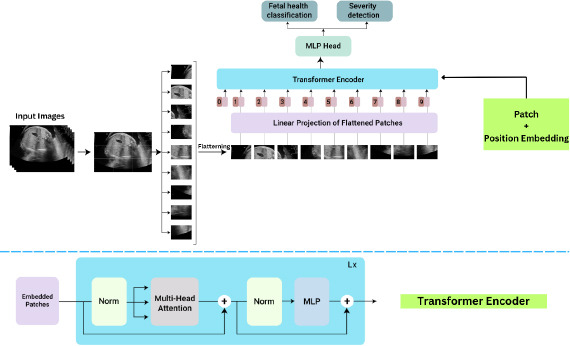
Transformer-based architecture for fetal health classification and severity detection.

**Fig. (12):**
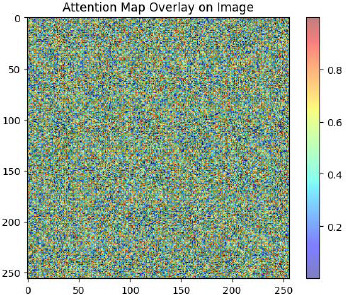
Attention map overlay on the input image for analysis.

**Fig. (13):**
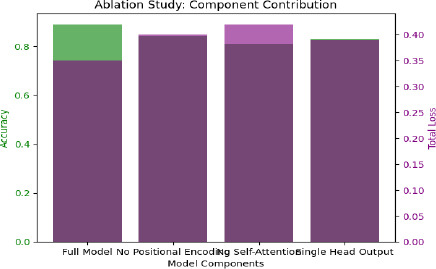
Ablation study comparing the accuracy and total loss across model components.

**Fig. (14):**
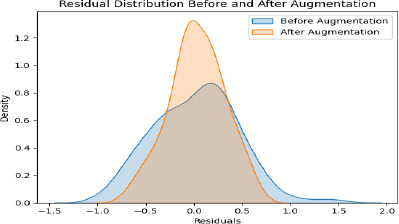
Residual distribution comparison before and after data augmentation.

**Fig. (15):**
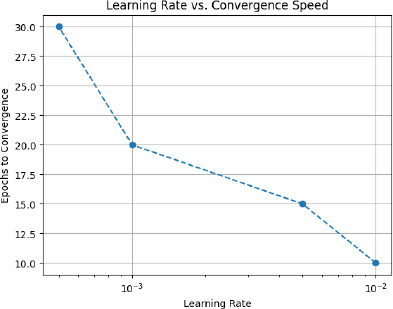
Relationship between learning rate and convergence speed in training.

**Fig. (16):**
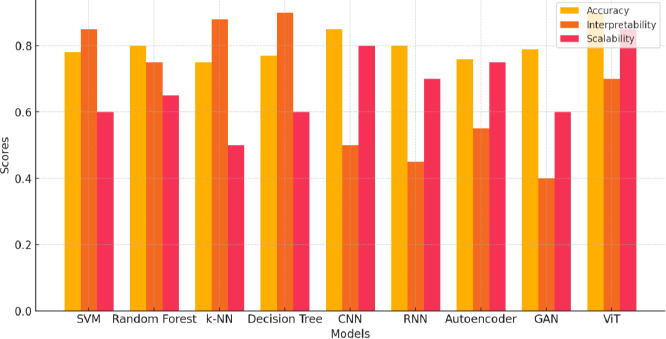
Comparison of fetal health assessment models on accuracy, interpretability, and scalability.

**Table 1:** Image augmentation techniques applied during model training.

Augmentation Step	Transformation Applied	Example Parameters	Input Image Size (H, W)	Output Image Size (H, W)	Description	Visual Representation
Original Image	-	-	(256, 256)	(256, 256)	Original input image before any augmentation is applied.	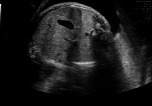
Random Rotation	Rotation by a random angle θ	θ=20^∘^	(256, 256)	(256, 256)	The image rotated by 20 degrees. The image remains in the same resolution.	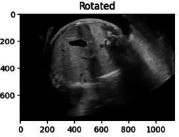
Random Horizontal Flip	Horizontal flip with probability *p*	*p*=0.5	(256, 256)	(256, 256)	Image flipped horizontally with 50% probability.	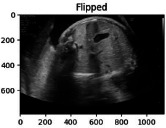
Random Zoom	Zoom by a factor *z*	*z*=1.1	(256, 256)	(282, 282) (cropped back to 256x256)	Image zoomed in by 10%, potentially requiring cropping back to the original size.	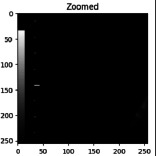
Random Shift	Shift image by Δx and Δy pixels	Δx=10, Δy=−5	(256, 256)	(256, 256)	The image shifted 10 pixels to the right and 5 pixels up.	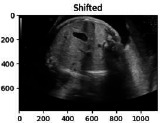
Brightness Adjustment	Adjust brightness by factor β	β=1.2	(256, 256)	(256, 256)	Brightness increased by 20%.	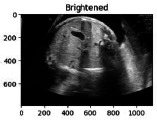
Contrast Adjustment	Adjust the contrast by factor γ	γ=1.5	(256, 256)	(256, 256)	Contrast enhanced by 50%.	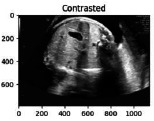

**Table 2:** Output for patch-based embedding.

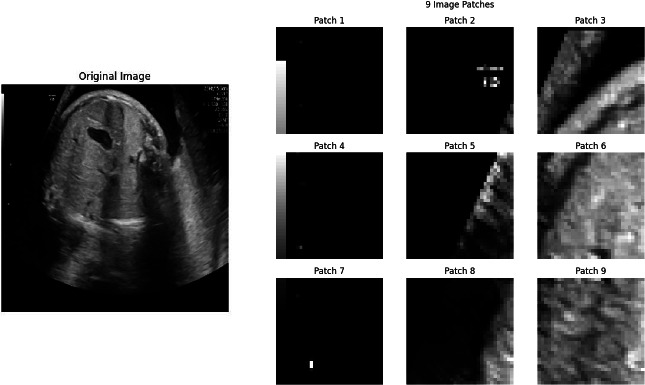

**Table 3:** Mathematical formulations for total loss, classification, and severity loss.

**Component**	**Mathematical Formulation**	**Visualizations**
Total Loss L_total_	L_total_​=L_class_​+λL_severity_	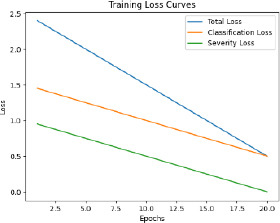
Classification Loss L_class_	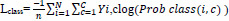	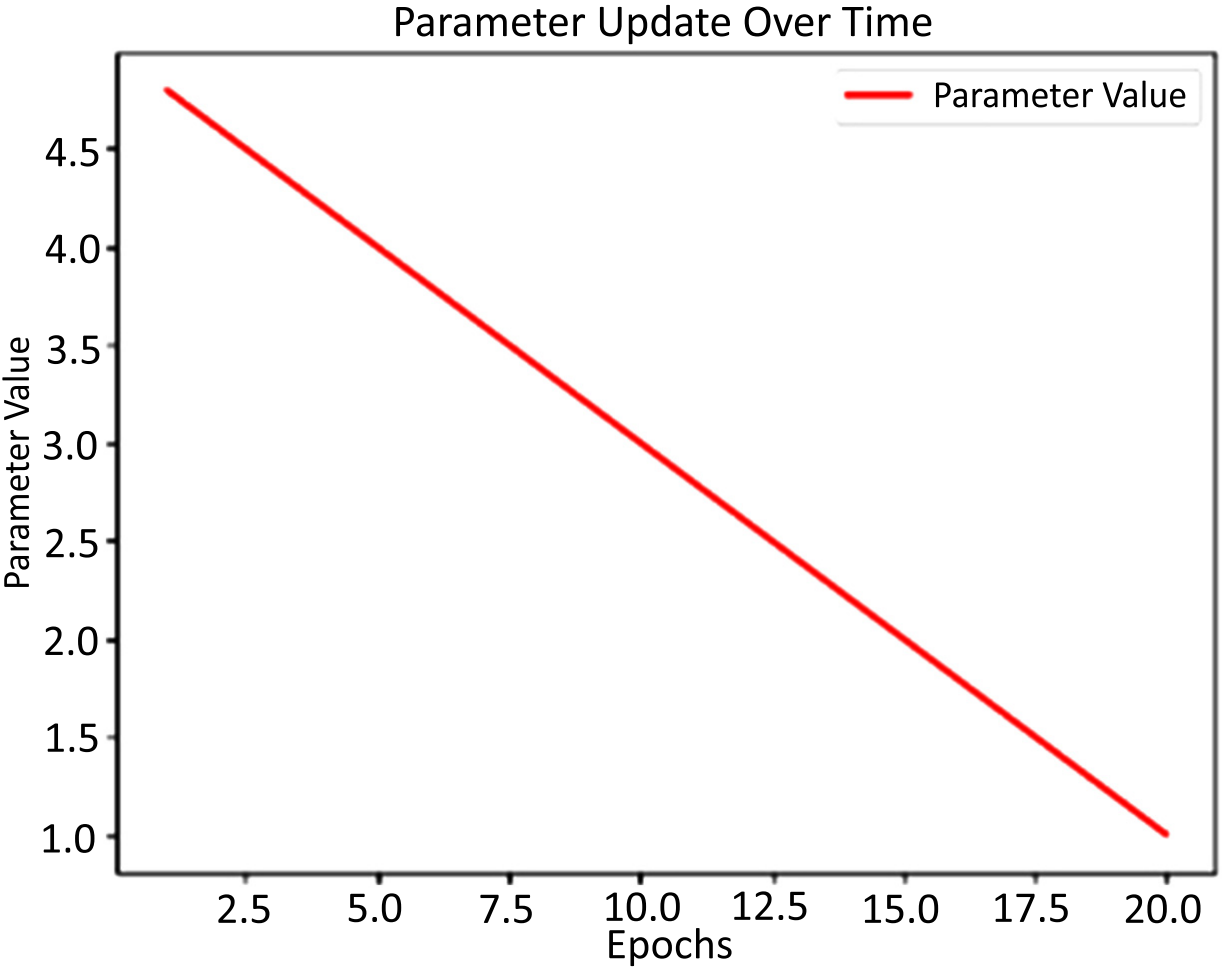
Severity Loss L_severity_	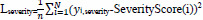	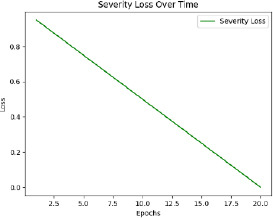
Parameter Update Rule	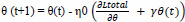	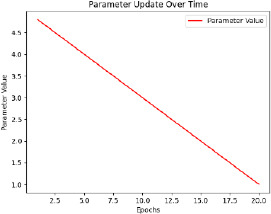

**Table 4:** Performance metrics comparison between training and validation sets.

**Metric**	**Training Set Value**	**Validation Set Value**	**Visualization**
**Total Loss**	0.35	0.45	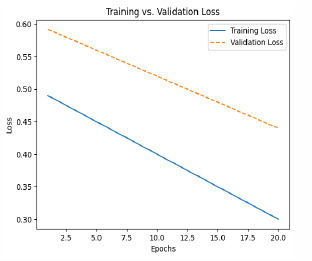
**Classification Accuracy**	92%	89%	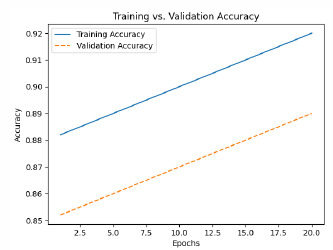
**Precision**	0.91	0.88	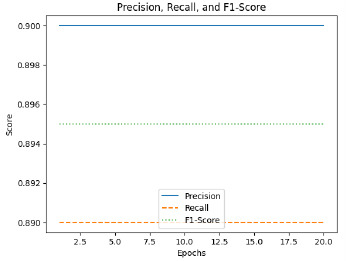
**Recall**	0.90	0.87	
**F1-Score**	0.905	0.875	
**Mean Absolute Error (MAE)**	0.25	0.30	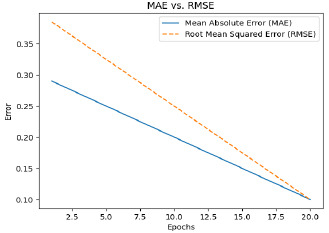
**Root Mean Squared Error (RMSE)**	0.35	0.40	

**Table 5:** Quantitative Results for Attention Map Analysis Across Severity Classes

** Severity Class **	** CoG Shift (pixels) **	** AFR (%) **	** WRS (Normalized) **	** AED (%) **	** ODM (%) **
** Mild **	4.2	65	0.72	20	68
** Moderate **	3.8	72	0.81	15	75
** Severe **	2.5	78	0.89	10	80

**Table 6:** Performance comparison before and after attention map optimization .

**Severity Class**	**Metric**	**Before Optimization**	**After Optimization**	**Improvement (%)**
**Mild**	IoU (%)	65	70	+7.7
	DSC (%)	78	82	+5.1
	Precision (%)	80	85	+6.3
	Recall (%)	75	80	+6.7
	F1-Score (%)	77	82	+6.5
**Moderate**	IoU (%)	68	72	+5.9
	DSC (%)	80	84	+5.0
	Precision (%)	83	87	+4.8
	Recall (%)	77	83	+7.8
	F1-Score (%)	80	85	+6.3
**Severe**	IoU (%)	72	75	+4.2
	DSC (%)	83	86	+3.6
	Precision (%)	85	89	+4.7
	Recall (%)	81	85	+4.9
	F1-Score (%)	83	87	+4.8

**Table 7:** Ablation study results showing accuracy and total loss comparison.

Component	Accuracy	Total Loss
Full model	0.89	0.35
No positional encoding	0.84	0.40
No self-attention	0.81	0.42
Dual head output	0.83	0.39

**Table 8:** Comparison of MAE and RMSE for training and validation sets.

Metric	Training Set Value	Validation Set Value
MAE	0.25	0.30
RMSE	0.35	0.40

**Table 9:** Learning rate versus epochs to model convergence comparison.

Learning Rate	Epochs to Convergence
0.01	10
0.005	15
0.001	20
0.0005	30

**Table 10:** Precision, recall, F1-score, and overall classification metrics comparison.

**Metric**	**Class 0**	**Class 1**	**Class 2**	**Overall**
Precision	0.75	1.00	1.00	0.93 (Weighted)
Recall	1.00	0.67	1.00	0.90 (Weighted)
F1-Score	0.86	0.80	1.00	0.90 (Weighted)
Support	3	3	4	10
Accuracy	-	-	-	0.90
Macro Average	0.92	0.89	0.89	0.90 (Average)
Cohen's Kappa	-	-	-	0.83

**Table 11:** Performance comparison between original and augmented data sets.

**Metrics**	**Original Data (500 Images)**	**Augmented Data (2500 Images)**
**Accuracy**	0.88	0.90
**Precision**	0.85	0.88
**Recall**	0.84	0.87
**F1-Score**	0.85	0.88
**MAE**	0.22	0.2
**Training Time (Epochs)**	10.0	8.0
**Validation Loss**	0.15	0.12
**Validation Accuracy**	0.88	0.90

**Table 12:** Confidence Intervals for Performance Metrics

**Metric**	**Original Data (95% CI)**	**Augmented Data (95% CI)**
**Accuracy**	86.18% – 89.02%	89.18% – 92.02%
**Precision**	84.00% – 86.00%	87.00% – 89.00%
**Recall**	83.00% – 85.00%	86.00% – 88.00%
**F1-Score**	84.00% – 86.00%	87.00% – 89.00%
**MAE**	0.21 – 0.23	0.19 – 0.21

**Table 13:** Effect Sizes (Cohen’s d) for Performance Improvements

**Metric**	**Effect Size (Cohen's d)**	**Interpretation**
**Accuracy**	2.63	Large
**Precision**	2.40	Large
**Recall**	2.31	Large
**F1-Score**	2.50	Large
**MAE**	1.45	Large

**Table 14:** Quantifying the Impact of Data Augmentation on Model Performance Using Cohen’s d

Metric	Before Augmentation	After Augmentation	Percentage Change	Cohen's d
Mean Accuracy	87.6%	90.8%	+3.65%	3.37
Standard Deviation	1.1%	0.8%	-27.3%	

**Table 15:** Impact of Weighting Parameter (λ) on Classification and Severity Detection Accuracy

λ	Classification Accuracy (%)	Severity Detection Accuracy (%)	Classification Change (%)	Severity Change (%)
0.1	85.2	91.6	0%	0%
0.3	88.4	89.2	+3.76%	-2.62%
0.5	91.0	85.8	+6.81%	-6.34%
0.7	92.2	82.4	+8.22%	-10.04%
0.9	93.1	78.2	+9.28%	-14.63%

**Table 16:** State-of-the-art comparison of model performance metrics across various studies and the proposed model.

Paper (Year)/Refs.	Accuracy (%)	Precision (%)	Recall (%)	F1-Score
Meng *et al.* (2020) [31]	81	78	77	77
Montero *et al.* (2021) [32]	81	-	-	80
Chen *et al.* (2017) [33]	87	71	64	64
Pu *et al.* (2021) [34]	85	85	85	85
Lee *et al.* (2021) [35]	75	73	73	72
Dong *et al.* (2019) [36]	-	-	-	-
Lin *et al.* (2018) [13]	79	85	-	-
Baumgarmer *et al.* (2017) [37]	77	90	90	-
Yaqub *et al.* (2017) [38]	85	-	-	-
Schlemper *et al.* (2019) [39]	89	83	80	81
Burgos-Artizzu *et al.* (2020) [40]	88	86	85	85
Sundaresan *et al.* (2017) [41]	84	80	79	79
Tan *et al.* (2019) [42]	88	87	86	85
Cai *et al.* (2020) [43]	88	85	83	84
Qu *et al.* (2020) [44]	89	86	85	85
Dou *et al.* (2019) [45]	88	86	85	85
Liang *et al.* (2019) [46]	89	87	86	85
Gao *et al.* (2020) [47]	88	86	85	85
Komatsu *et al.* (2021) [48]	85	84	83	83
Zhang *et al.* (2021) [49]	89	85	85	84
Proposed Model	91	93	90	90

## Data Availability

The data and supportive information are available within the article.

## LIST OF ABBREVIATIONS

ViTs: Vision Transformers
CNNs: Convolutional Neural Networks
RNNs: Recurrent Neural Networks
SVMs: Support Vector Machines
RFs: Random Forests
